# Differential Diel Translation of Transcripts With Roles in the Transfer and Utilization of Iron-Sulfur Clusters in Arabidopsis

**DOI:** 10.3389/fpls.2018.01641

**Published:** 2018-11-13

**Authors:** Hongliang Zhang, Ute Krämer

**Affiliations:** Molecular Genetics and Physiology of Plants, Faculty of Biology and Biotechnology, Ruhr University Bochum, Bochum, Germany

**Keywords:** translatome, microarray, diurnal, metalloprotein, Fe, Zn, Mn, Cu

## Abstract

Iron-sulfur (Fe-S) clusters are evolutionarily ancient ubiquitous protein cofactors which have mostly catalytic functions but can also have structural roles. In *Arabidopsis thaliana*, we presently know a total of 124 Fe-S metalloproteins that are encoded in the genome. Fe-S clusters are highly sensitive to oxidation. Therefore, we hypothesized that Fe-S cluster protein biogenesis is adjusted following the daily rhythms in metabolism driven by photosynthesis at the whole-plant, organ, cellular and sub-cellular levels. It had been concluded previously that little such regulation occurs at the transcript level among the genes functioning in Fe-S cluster assembly. As an initial step toward testing our hypothesis, we thus addressed the diel time course of the translation state of relevant transcripts based on publicly available genome-wide microarray data. This analysis can answer whether the translation of the pool of transcripts of a given gene is temporarily either enhanced or suppressed, and when during the day. Thirty-three percent of the transcripts with functions in Fe-S cluster assembly exhibited significant changes in translation state over a diurnal time course, compared to 26% of all detected transcripts. These transcripts comprised functions in all three steps of cluster assembly including persulfide formation, Fe-S cluster formation and Fe-S cluster transfer to target apoproteins. The number of Fe-S cluster carrier/transfer functions contributed more than half of these transcripts, which reached maxima in translation state either during the night or the end of the night. Similarly, translation state of mitochondrial frataxin and ferredoxin, which are thought to contribute Fe and electrons during cluster formation, peaked during the night. By contrast, translation state of chloroplast SUFE2 in persulfide formation and cytosolic Fe-S cluster formation scaffold protein NBP35 reached maxima in translation state during the day. Among the transcripts encoding target Fe-S cluster-utilizing proteins, 19% exhibited diurnal variation in translation state. Day-time maxima of translation state were most common among these transcripts, with none of the maxima during the night (ZT18). We conclude that diurnal regulation of translation state is important in metalloprotein biogenesis. Future models of Fe-S protein biogenesis require more comprehensive data and will have to accommodate diurnal dynamics.

## Introduction

Across all groups of biological organisms, proteins that can bind one or several iron-sulfur (Fe-S) cluster cofactors *in vivo* (Fe-S proteins) fulfill central cellular biochemical functions. Several types exist of protein-bound Fe-S clusters, based on chemical structures and oxidation states. Among these, cubane [4Fe–4S] and rhombic [2Fe–2S] are the most widespread Fe–S clusters, containing ferrous or ferric Fe^(+II)^ or ^(+III)^ and sulfide S^(-II)^ (Lill, 2009; Balk and Schaedler, 2014). Disruption of Fe-S protein biogenesis has been associated with serious diseases in human and animals (Lill, 2009; Wachnowsky et al., 2017). For example, frataxin is thought to function as the iron donor for the mitochondrial Isu1 scaffold protein complex in Fe-S cluster assembly in human. Depletion of frataxin causes the neurodegenerative disease Friedreich’s ataxia, attributed to defective Fe–S protein activity and iron accumulation (Campuzano et al., 1996; Rötig et al., 1997).

In plants, Fe-S proteins are abundant in mitochondria and chloroplasts, for example in respiratory and photosynthetic electron transfer chains (Blaby-Haas and Merchant, 2013; Couturier et al., 2013), and Fe-S cluster assembly occurs in both of these organelles (Balk and Schaedler, 2014). Notably, the metabolism, repair and epigenetic modification of DNA all depend on Fe-S protein functions (Buzas et al., 2014; Zhang, 2015). The catalytic subunits of eukaryotic DNA polymerases α, δ, 𝜀, and ζ contain [4Fe-4S] cofactors that are required for protein-protein interactions in the active protein complex (Netz et al., 2012). In Arabidopsis, Fe-S cluster-binding residues are conserved in these proteins, namely POLA1, POLD1, POLE1A/POL2A, POLE1B/POL2B, and REV3, the catalytic subunit of DNA polymerase ζ (Shultz et al., 2007; Supplementary Dataset 1). Moreover, proteins of the Demeter (DME) family contain a [4Fe-4S] cofactor and function in the demethylation and base excision repair of DNA (Choi et al., 2002; Wöhrmann et al., 2012; Buzas, 2016). Expanding from the list of Fe-S proteins updated in 2010 (Balk and Pilon, 2011), a total of 124 genes encoding proteins that contain Fe-S clusters are presently known in Arabidopsis (Supplementary Dataset 1).

**FIGURE 1:**
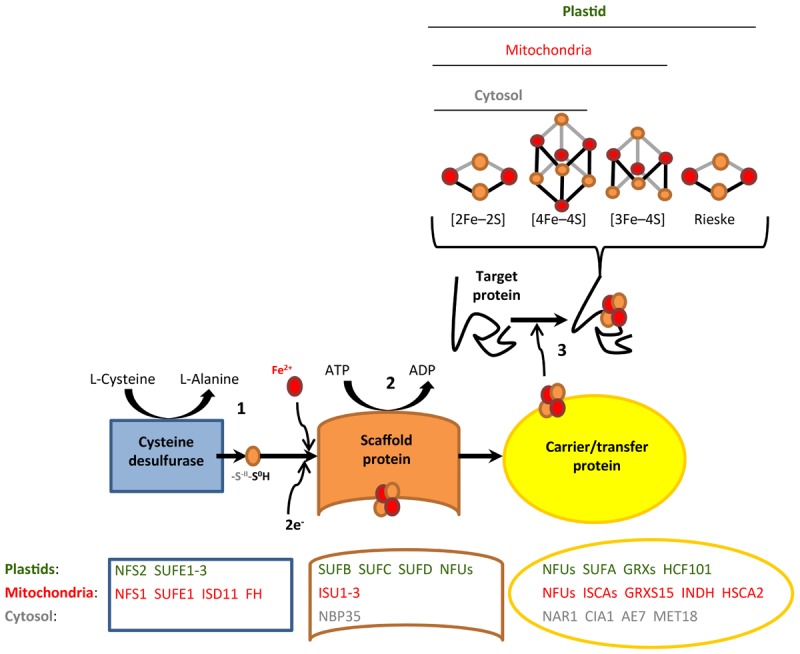
Generalized scheme of Fe-S cluster assembly. Shown are the three consecutive steps of persulfide formation **(1)**, Fe-S cluster formation on the scaffold protein **(2)**, and Fe-S cluster transfer to target apoproteins *via* carrier/transfer proteins **(3)**. Major known and predicted Fe-S cluster assembly proteins are listed. The identification of a number of additional putative assembly proteins was based on studies in yeast or human cells and the presence of homologous candidate genes in the *Arabidopsis* genome (see Supplementary Dataset 2).

The biosynthesis of Fe-S proteins requires cellular Fe-S cluster assembly. The first knowledge on Fe-S assembly pathways was obtained in *Escherichia coli* and *Azotobacter vinelandii* (Lill, 2009). Subsequent work revealed that genes encoding Fe-S assembly proteins are well conserved from prokaryotes to eukaryotes (Balk and Lobréaux, 2005; Lill, 2009; Py and Barras, 2010). In recent years, an increasing number of proteins of the Fe-S cluster assembly pathways were characterized employing mutants of *A. thaliana* (Couturier et al., 2013; Balk and Schaedler, 2014), and yet, our present knowledge in plants still relies to a large degree on inference from other model organisms. In plants, there are three cellular pathways of Fe-S cluster biogenesis. These are the Sulfur mobilization (SUF) pathway in plastids, the Iron Sulfur Cluster (ISC) pathway in mitochondria, and Cytosolic Iron-sulfur protein Assembly (CIA) pathway in the cytosol. Although each of these Fe-S cluster assembly pathways involves different proteins, all three of them proceed according to common biosynthetic principles (Figure 1). First, cysteine is metabolized by cysteine desulfurase to generate a persulfide (protein-S-S-H) at the active site of the enzyme. Subsequently, a terminal sulfane S^0^ of this persulfide is transferred to a scaffold protein complex, followed by the reduction to sulfide (S^-II^). The integration of iron on the scaffold protein results in the formation of the Fe-S cluster. Finally, the assembled Fe-S cluster is transferred to target apoproteins via carrier/transfer proteins. Based on three excellent reviews (Balk and Pilon, 2011; Couturier et al., 2013; Balk and Schaedler, 2014), we have assembled a list of 49 *A. thaliana* genes encoding proteins involved in Fe-S cluster assembly (Supplementary Dataset 2). Note that some of these Fe-S cluster assembly proteins bind Fe-S clusters themselves, mostly Fe-S scaffold and carrier/transfer proteins.

Although we have good knowledge of the Fe-S protein biogenesis machinery, we know comparably little about the regulation of Fe-S protein biogenesis. The Fe-S protein biogenesis machinery of bacteria involves transcriptional regulators, such as Fur, OxyR and IscR. For example, under oxidative stress, OxyR activates the expression of the *suf* operon in *E. coli* (Roche et al., 2013). However, none of the known bacterial regulators of Fe-S protein biogenesis has any orthologs in a plant or an algal genome (Couturier et al., 2013), suggesting that plants utilize different regulatory mechanisms. Protein-bound Fe-S clusters can be destroyed by oxygen or reactive oxygen species (ROS), such as hydrogen peroxide or nitric oxide (Beinert et al., 1997). Free Fe^2+^ released from Fe-S clusters can catalyze the Fenton reaction, which generates even more ROS (Halliwell and Gutteridge, 1992; Ravet et al., 2009). Transcript levels of few genes encoding Fe–S cluster assembly proteins respond to oxidative stress, nutritional iron deficiency, and heavy metal excess, in plants (Liang et al., 2014) (see also Discussion). Overall there is only little environment-dependent regulation in plants of Fe-S cluster assembly at the transcript level (Balk and Schaedler, 2014).

In plants, contrasting photosynthetic activity between day and night confers a diel rhythm in plant metabolism as well as in the generation of ROS, primarily locally in chloroplasts but also across cells, tissues and organs of plants (Lai et al., 2012; Sierla et al., 2013; Trotta et al., 2014). Plants are capable of regulatory adjustment to these fluctuations, which can be controlled by light, stress, metabolic state or the circadian clock (Lai et al., 2012**;** Sierla et al., 2013; Trotta et al., 2014). Surprisingly, it was noted that Fe-S cluster assembly – a potentially highly ROS-sensitive process – exhibits no diurnal profile of regulation at the transcript level (Balk and Schaedler, 2014). Consequently, Fe-S protein biogenesis of plants is either sufficiently ROS-insensitive, or alternatively it could be diurnally controlled at a different level of regulation.

To examine whether Fe-S protein biogenesis is diurnally regulated at the level of translational regulation, we re-analyzed published microarray-based diurnal translatome data (Missra et al., 2015). Accordingly, subsets of Fe-S cluster assembly and Fe-S utilizing proteins are under pronounced translational control. Among these, the translation of transcripts encoding Fe-cluster assembly proteins tended to peak at night while the translation of the majority of transcripts encoding Fe cluster-utilizing proteins reached a minimum. We conclude that the diurnal regulation of metalloprotein biogenesis deserves to be addressed in a more quantitative and comprehensive translatome sequencing approach.

## Materials and Methods

**FIGURE 2:**
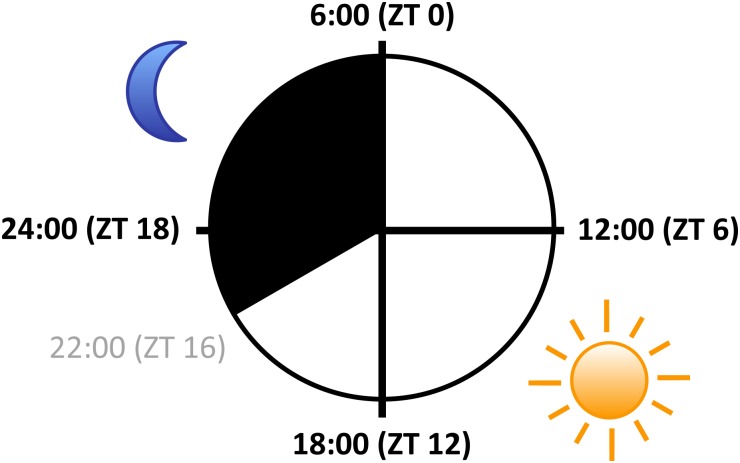
Sampling scheme over the diurnal cycle. *Arabidopsis* seedlings were grown on agar-solidified full-strength MS media in petri dishes at constant temperature of 22°C in 16 h light (white; light intensity 80 μmol m^-2^ s^-1^)/8 h dark (black) cycles for 10 days, and harvested at ZT 0, ZT 6, ZT 12, and ZT 18, respectively (black, bold fonts; ZT: Zeitgeber Time). Diagram represents the sampling design of Missra et al. (2015).

Diel translatome time course data from ATH1 GeneChip microarray hybridization was taken from Missra et al. (2015) (Figure 2). Briefly, RNA was fractionated into non-polysomal (NP), small polysomal (SP), and large polysomal (LP) fractions using sucrose density centrifugation. In these fractions, the mRNA molecules were estimated to be bound by two and seven ribosomes on average so that SP and LP fractions were weighted by 2 and 7, respectively (Missra et al., 2015). The translation state (TL) of a given mRNA was approximated as follows: TL = (2 × SP + 7 × LP)/(NP + SP + LP), resulting in a global maximum TL of 6.48 and a minimum TL of 0.46. We additionally estimated relative rates of protein biosynthesis (PBR) for each protein across the diel time course by multiplying translation state with transcript levels (PBR = TL× TX, whereby TX = NP + SP + LP) as given in Missra et al. (2015). This calculation is based on the assumption that there is a correlation between the amount of ribosome-associated mRNA and its rate of translation, which is a simplification. The authors made no adjustment for global oscillation of TL across all expressed genes, which peaked at noon (ZT6; ∼ 0.8) and was minimal at the end of the night (ZT0; ∼ 0.68). Following the strategy employed by Missra et al. (2015), transcripts concluded to exhibit diel variation of TL fulfilled one of three criteria: ΔTL (difference between maximum and minimum average TL of three replicates) > 0.7, or ANOVA (Analysis Of Variance) *P* < 0.05, or Significance Analysis of Microarrays (SAM) with a collective FDR < 0.10 (Tusher et al., 2001).

For analyzing the TL of Fe-S proteins and Fe-S assembly proteins in this study, we first assembled a list of 124 Fe-S cluster-containing proteins (Supplementary Dataset 1) and 49 Fe-S assembly proteins (Supplementary Dataset 2) identified in *A. thaliana* so far, expanding from previous work (Balk and Pilon, 2011; Couturier et al., 2013; Balk and Schaedler, 2014). Then, the translatome data of the genes in the two lists of Fe-S proteins and Fe-S assembly proteins were extracted from Supplementary Dataset 1 of the original publication (Missra et al., 2015) as shown in Supplementary Datasets 3, 4 of this publication. Note that we consider as Fe-S cluster-utilizing proteins (Supplementary Dataset 4) only those Fe-S cluster-containing proteins that are not involved in Fe-S cluster assembly (Supplementary Dataset 3). Translatome data were further analyzed by using the software Genesis 1.7.6 (Sturn et al., 2002). First, translatome data were scaled by the mean center method. That is, for each transcript at each time point, *Z* = TL_ZT0,6,12,or18_ – average (TL_ZT0_ + TL_ZT6_ + TL_ZT12_ + TL_ZT18_). The purpose of this was to aid visualization of diel cycles of a number of genes together. Subsequently, the transcripts were clustered into different groups by the average linkage clustering method (Sokal and Michener, 1958). Analysis of TL of metalloproteins dependent on cofactors other than Fe-S clusters was done based on manually assembled lists (Supplementary Dataset 5). Only the transcripts showing significant diurnal dynamics at the translatome level were used when calculating the proportion of transcripts reaching maximum TL at each time point.

## Results

### Global Translation Status of Fe-S Proteins and Fe-S Assembly Proteins Over a Diurnal Cycle

According to the previous analysis, out of 12,342 detected transcripts, 3,218 transcripts (26%) varied significantly in their translation state (TL) over a diurnal cycle (Supplementary Dataset 6). In this dataset, 39 (out of a total of 49) transcripts encoding Fe-S assembly proteins and 68 (out of 97) transcripts encoding Fe-S cluster-utilizing proteins were detected as expressed (Supplementary Datasets 3, 4). Among these, 13 transcripts encoding Fe-S assembly proteins (33%) and 13 transcripts encoding Fe-S cluster-utilizing proteins (19%) showed diurnal fluctuations in TL (Table 1). The apparent over-representation of Fe-S cluster assembly genes among genes with diurnal changes in TL was not statistically significant according to a Fisher’s exact test.

**Table 1:** Extent of diurnal regulation of translation state of Fe-S cluster-related proteins.

	Transcripts detected^a^ (number of genes)	Diurnal variation in Translation State (TL)^b^ (number of genes)
Fe-S cluster assembly	39	13 (33%)
Fe-S cluster use	68	13 (19%)
All functions	12,342	3,218 (26%)


*^*a*^Transcript levels detected as present in all ribosome-associated fractions at all four time points (Missra et al., 2015).^*b*^Number counts for transcripts exhibiting significant diurnal variation in TL according to Missra et al. (2015) (see Materials andMethods).*

### Transcripts Encoding Fe-S Assembly Proteins With Diurnal Changes in Translation State

**FIGURE 3:**
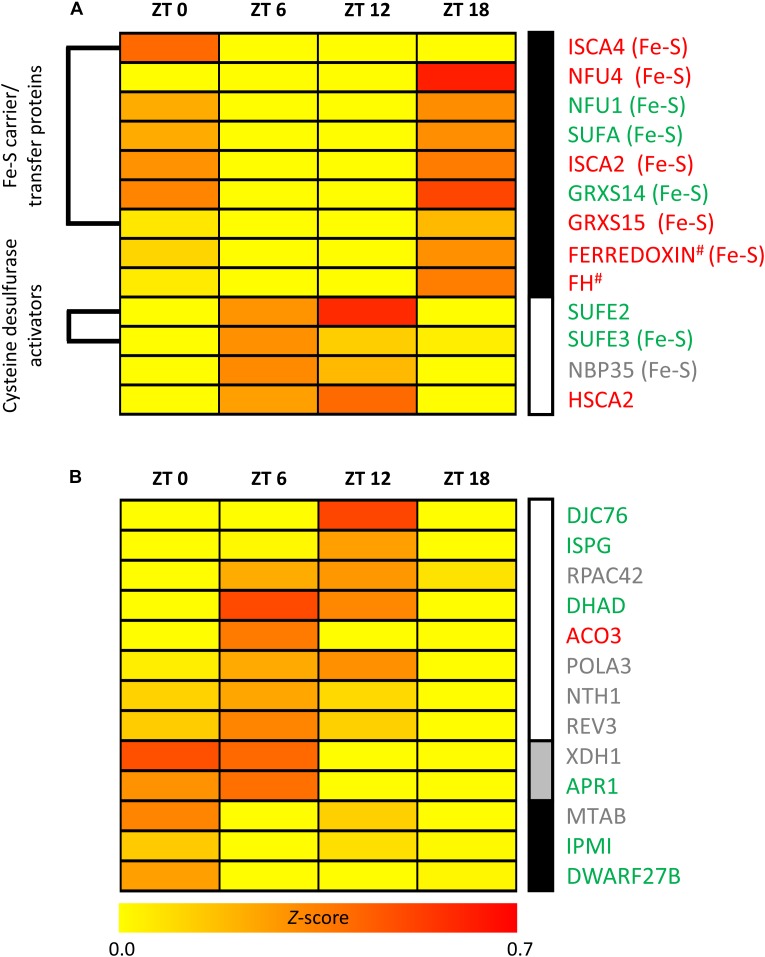
Transcripts encoding Fe-S cluster-related proteins that exhibit significant diurnal variation in translation state. **(A)** Fe-S cluster assembly, **(B)** Fe-S cluster utilization. TL data, scaled by the mean center method for each sampling time point, were analyzed by software Genesis 1.7.6. Subsequently, the identified groups were subjected to average linkage clustering. Clusters are marked by differently shaded vertical bars (right). The translation state represents an estimate of ribosome occupancy per transcript. #Putative assembly protein. Fe-S: indicates the ability to bind an Fe-S cluster in **(A)**.

Thirteen transcripts encoding Fe-S assembly proteins varied significantly in their TL over the diurnal cycle. Among these 13 transcripts, 7 correspond to mitochondrial, 5 to plastidic and one to cytosolic proteins. These transcripts clustered into two groups of similar TL profiles over the diurnal cycle (Figure 3A). TL of transcripts in the first cluster peaked during and at the end of the night (ZT18 or ZT0). Interestingly, 7 out of 9 transcripts in this group encode proteins associated with Fe-S cluster carriage/transfer, including mitochondrial ISCA2, ISCA4, NFU4, and GRXS15 and chloroplast SUFA, NFU1 and GRXS14. The remaining two transcripts in this cluster encode the candidate Fe donor protein mitochondrial frataxin (FH) and the alpha-helical mitochondrial ferredoxin, respectively. This ferredoxin protein is thought to provide an electron for Fe-S cluster synthesis on ISU1, the major scaffold protein in mitochondria. TL of transcripts in the second cluster peaked during the day (ZT6 or ZT12). Among the four transcripts that reached peak TL during the day, two encode plastidic cysteine desulfurase activators and interactors of CpNifS, namely SUFE2 and SUFE3 (Narayana Murthy et al., 2007). The other two transcripts encode the cytosolic Fe-S assembly scaffold protein NBP35 (Bych et al., 2008; Kohbushi et al., 2009; Bastow et al., 2017), and HSCA2, one of two abundant mitochondrial HSP70-type chaperones which was proposed to function in Fe-S cluster transfer (Balk and Pilon, 2011). The estimation of protein biosynthesis rates (PBR) (Supplementary Figure 1A and Supplementary Dataset 7) resulted in diel profiles that were similar to TL for most Fe-S assembly proteins, but considerably more diverse than diel TL profiles for Fe-S carrier/transfer proteins (see Figure 3A).

### Transcripts Encoding Fe-S Proteins With Diurnal Changes in Translation State

Twenty-three transcripts encoding Fe-S proteins varied significantly in TL over the diurnal time course. Among these, 13 transcripts encode Fe-S cluster-utilizing proteins (Figure 3B) and 10 transcripts encode Fe-S proteins that are involved in cluster assembly and Fe-S protein biogenesis (Figure 3A). The 13 transcripts encoding Fe-S cluster-utilizing proteins comprised 6 plastidic, 4 nuclear, 2 cytosolic, and 1 mitochondrial localized proteins. Proteins detected to exhibit diurnal regulation of TL have various functions, in particular DNA and RNA metabolism as well as isoprenoid and leucine biosynthesis. All except two of these proteins are predicted to bind the most abundant [4Fe-4S] cluster, which is less stable than the [2Fe-2S] cluster. Only DWARF27B, a homolog of a rice β-carotene isomerase protein acting in strigolactone biosynthesis, and Xanthine the dehydrogenase XDH1 bind [2Fe-2S] clusters (Zhang et al., 2012; Balk and Schaedler, 2014). Fe-S cluster-utilizing proteins fell into three groups of differing diel TL profiles (Figure 3B). TL of 8 transcripts peaked during the day (ZT6 or ZT12), TL of two transcripts peaked at ZT0 and ZT6, and TL of three transcripts peaked at the end of the night (ZT0) (Figure 3B). TL of none of the Fe-S cluster-utilizing proteins peaked at ZT18 during the night, in contrast to the Fe-S carrier/transfer proteins that exhibited diel TL differences (see Figure 3A). When, instead of TL, we considered estimates of PBR, we mostly observed no changes or only minor shifts for few proteins (Supplementary Figure 1 and Supplementary Dataset 8).

Among the transcripts showing diel changes in TL, ten transcripts encode Fe-S proteins that are also involved in Fe-S protein biogenesis (see Figure 3A). The TL of 7 of these 10 transcripts peaked during the night (ZT18) and one peaked at the end of the night (ISCA4). Only the cytosolic Fe-S cluster scaffold protein NBP35 and SUFE3 peaked during the daytime.

### Transcripts Encoding Proteins Binding Heme and Cationic Fe, Zn, Cu, or Mn With Diurnal Changes in Translation State

**FIGURE 4:**
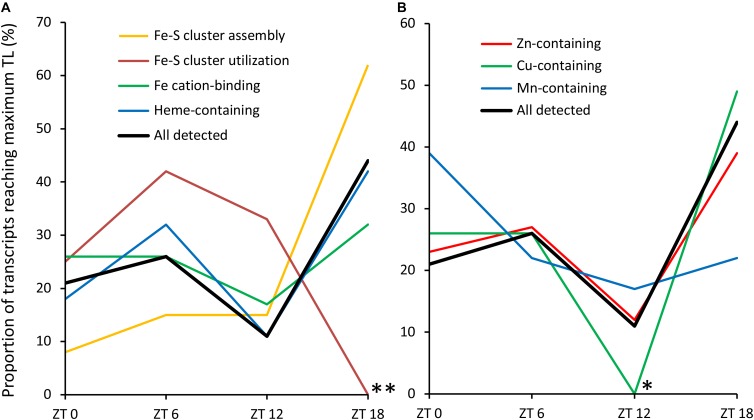
Diurnal TL maxima of transcripts encoding metalloproteins. **(A)** Fe-dependent metalloproteins. **(B)** Other metalloproteins. Only the transcripts showing significant diurnal variation in TL were used in this analysis (see Tables 1, 2). Asterisks mark significant differences from all detected transcripts at the same time point (Fisher’s exact test; ^∗^*P* < 0.05, ^∗∗^*P* < 0.01).

To compare Fe-S cluster-containing proteins with other metalloproteins, we determined the proportion showing diurnal variation in TL among proteins binding heme/cytochrome cofactors (29%) and proteins binding cationic Fe (28%), Zn (22%), Cu (30%), or Mn (39%) (Table 2). Among these, in comparison to all functions (26%) only Zn-dependent proteins are significantly differently represented, with an underrepresentation in contrast to the trend for over-representation of heme/cytochrome-dependent, Cu-dependent and Mn-dependent proteins. In order to compare the timing of biogenesis of metalloproteins among those exhibiting significant variation in TL over the diurnal cycle, we determined the proportion of transcripts peaking in TL at each time point (Figure 4). Whereas TL of 62% of transcripts encoding Fe-S cluster assembly proteins peaked at ZT18, TL of none of the transcripts encoding Fe-S cluster-utilizing proteins peaked at this time point (Figure 4A; statistically significantly different from all detected transcripts at *P* < 0.01, Fisher’s exact test), in accordance with observations described earlier (see Figure 3). The diel TL profile of transcripts encoding heme/cytochrome- as well as Fe, Zn, and Cu cation-binding proteins was similar to that of all detected transcripts, with a maximum of 30–50% peaking at ZT18, a less pronounced secondary maximum of 25–42% peaking at ZT6 and a minimum of 0–17% peaking at ZT12 (Figures 4A,B). Notably, none of the transcripts encoding Cu-dependent proteins showed an afternoon peak in TL at ZT12 (statistically significantly different from all detected transcripts, *P* < 0.05, Fisher’s exact text), and as many as 50% peaked during the night at ZT18. The diel course of TL of transcripts encoding Mn-dependent proteins differed from that of all others, with a maximum of 39% of proteins peaking at the end of the night (ZT0).

**Table 2:** Extent of diurnal regulation of translation state of heme/cytochrome and Fe, Zn, Cu, and Mn cation-binding metalloproteins.

	Transcripts detected^a^ (number of genes)	Diurnal variation in Translation State (TL)^b^ (number of genes)
Fe cation-dependent	192	53 (28%)
Heme-dependent	255	74 (29%)
Zn-dependent	967	216 (22%)^∗^
Cu-dependent	131	39 (30%)
Mn-dependent	46	18 (39%)
All functions	12,342	3,218 (26%)


*^*a*^Transcript levels detected as present in all ribosome-associated fractions at all four time points (Missra et al., 2015).^*b*^Number counts for transcripts exhibiting significant diurnal variation in TL according to Missra et al. (2015) (see Materials andMethods). ^∗^P < 0.05 (Pearson Chi-Square test).*

## Discussion

Here we addressed the translation state of transcripts encoding Fe-S cluster-binding proteins and other metalloproteins over a diurnal cycle. While this has not been addressed to date, previous studies have addressed the responses of various components in Fe-S cluster assembly to changed environmental conditions.

Besides oxygen (see Introduction), iron and sulfur supply were reported to affect Fe-S cluster biosynthesis. Under iron deficiency conditions, *SUFB* expression is down-regulated in both *A. thaliana* (Xu et al., 2005; Sivitz et al., 2012; Rodríguez-Celma et al., 2013) and in rice (Liang et al., 2014). Corresponding to the decreased transcript level, SUFB protein abundance is also decreased (Hantzis et al., 2018). SUFB is a component of the SUFBC_2_D complex which functions as a scaffold protein in the plastidic SUF pathway (Hu et al., 2017). Besides SUFB, the protein abundance of a Fe-S carrier/transfer protein SUFA was decreased under Fe deficiency, whereas *SUFA* transcript abundance was unaffected, suggesting the possibility of regulation at the translational or protein level. Recently, it was reported that S and Fe uptake are closely coordinated (Astolfi et al., 2010; Astolfi et al., 2012; Zuchi et al., 2015). This is not surprising, given that Fe-S clusters are the most abundant among all the Fe-containing cofactors (Forieri et al., 2013; Balk and Schaedler, 2014; Vigani and Briat, 2016). Fe deficiency can result in the down-regulation of transcript levels of *sulfite reductase* (*SiR*). SiR is a protein involved in sulfate assimilation (Forieri et al., 2017), which contains an Fe_4_S_4_ cluster itself. This response might contribute to a decreased S flux into cysteine, the only S donor for Fe-S cluster biosynthesis (Balk and Schaedler, 2014).

Light is another environmental factor known to enhance the incorporation of Fe-S clusters into Fe-S cluster-utilizing proteins of isolated chloroplasts (Takahashi et al., 1986) as well as the expression of some Fe-S cluster assembly genes. *SUFE1* transcript levels were reported to increase 2.3-fold in the light (Ye et al., 2006), and light-grown plants contained 2.5 times higher SUFE1 protein levels than dark-grown plants. Another study showed that prolonged darkness leads to significant decreases in the protein abundance of several key components in SUF pathway, including GRXS14, NFU2, NFU3, SUFA, and SUFB (Rey et al., 2017).

It was reported that the expression of major Fe homeostasis genes is under circadian regulation, such as *IRT1*, *bHLH39*, and *FER1* (Hong et al., 2013). Regulation of ferritin gene (*AtFER1*, *AtFER3,* and *AtFER4*) expression by the clock was confirmed by showing that they are direct transcriptional targets of PRR7, a central clock component (Liu et al., 2013). In turn, Fe nutrition status can modulate the plant circadian clock, with period lengthening under Fe-deficient conditions (Chen et al., 2013; Hong et al., 2013; Salomé et al., 2013). We observed no significant diel regulation of translation state of these transcripts (Supplementary Dataset 9).

### TL of Transcripts Encoding Proteins in Early Steps of Fe-S Cluster Assembly and in Fe-S Cluster Transfer Show Contrary Diurnal Dynamics in Translation State

In our analysis, we separated Fe-S cluster-associated functions into Fe-S cluster assembly and Fe-S cluster utilization. By comparison to all genes detected as expressed, those with functions in Fe-S cluster assembly were slightly, ca. 1.3-fold overrepresented among the genes exhibiting diurnal variation in TL. By contrast, genes associated with Fe-S cluster utilization were slightly, ca. 0.7-fold, underrepresented (Table 1). Expanding this analysis, genes encoding Mn-dependent proteins were the most overrepresented, ca. 1.5-fold, among genes exhibiting diurnal variation in TL, followed by genes encoding Cu-dependent proteins (1.2-fold) (Table 2). The proportion of genes exhibiting diurnal variation in TL among heme/cytochrome-dependent and Fe cation-dependent proteins was similar to that of all expressed genes. It is important to note that none of these deviations were statistically significant, possibly because of a lack of sensitivity of statistical testing given the small number of genes in each of the functional classes. The only significant difference was an underrepresentation, approximately 0.85-fold, of genes encoding Zn-dependent proteins among those exhibiting significant diurnal variation in TL. The considerably larger number of genes in this class is likely to have facilitated the statistical detection of this effect. This result may reflect the high proportion of transcription factors of overall low abundance among Zn-dependent proteins, by comparison to genes encoding highly abundant proteins functioning in metabolism. Taking these results together, diel dynamics in TL are relevant for all classes of metalloproteins.

However, based on this preliminary analysis, the pervasiveness of such diel dynamics in TL among metalloproteins is similar or only slightly higher than for non-metalloproteins. Moreover, our results suggest differences between different types of metalloproteins and metalloprotein functions, with overall more pronounced diel dynamics in TL among Mn-dependent proteins and Fe-S cluster assembly proteins and overall less diel dynamics in TL among Zn-dependent proteins. Evidently, TL analyzed in this study cannot be seen as a proxy of the overall rate of protein biogenesis (PBR), which is better described by an estimate of the total amount of ribosomal occupancy on transcripts derived from a given gene in the future (e.g., in this study: PBR_est_ = 2 × SP + 7 × LP). A re-analysis of data shown in Figure 3 suggested little difference between the diel dynamics in TL and the diel dynamics in PBR (Supplementary Figure 1) except for Fe-S carrier/transfer proteins, for which diel PBR profiles were rather variable.

Among the metalloprotein-encoding genes exhibiting diurnal variation in TL, we observed some striking differences in the diel profiles (Figure 4). The deviating profiles for Mn and, very pronouncedly, Cu-dependent metalloproteins are consistent with an overall metal-specific differentiation of TL over the diel cycle for these metals. In addition, there was a clear difference between genes encoding Fe-S cluster assembly proteins and those encoding Fe-S cluster-utilizing proteins. More than 60% of the former peaked during the night at ZT18, whereas none of the latter peaked at this time point. Instead, TL of genes encoding Fe-S cluster-utilizing proteins peaked mostly ZT6 (42%) and ZT12 (33%). A possible interpretation of this observation is the occurrence of a temporal gap between the biogenesis of Fe-S clusters and their incorporation into freshly synthesized polypeptide chains of Fe-S cluster-utilizing apo-metalloproteins.

A more detailed examination of the diel TL profiles of Fe-S cluster assembly proteins identified two distinct clusters (Figure 3A). The Fe-S assembly proteins in the first cluster, for which TL peaked during the night (ZT18 and to a lesser degree ZT0) mainly participate in the third step of Fe-S cluster protein biogenesis, which is Fe-S cluster transfer to target apoproteins via carrier/transfer proteins (Figure 1). By contrast, the three Fe-S cluster assembly proteins SUFE2, SUFE3, and NBP35 in the second cluster, which peaked during the day (ZT12 and ZT6), contribute to the first two steps of Fe-S protein biogenesis, persulfide formation and Fe-S cluster formation on the scaffold protein complex (Figure 1). These data suggest that some functions in Fe-S cluster synthesis and Fe-S cluster transfer to apoproteins have opposing diel dynamics of association with ribosomes. Taking all our observations together, the biosynthesis of apometalloproteins that require Fe-S clusters appears to occur preferentially during the day, as well as just before dawn for some cytosolic/nuclear Fe-S proteins (Figure 3 and Supplementary Figure 1).

In contrast to cytoplasmic NBP35, the main scaffold proteins SUFB, C and D of plastids and ISU1 of mitochondria did not show diurnal dynamics at the level of translation state. In the context of the model described above, it is possible that their basal transcript levels and translation states are sufficiently high throughout the day, which can satisfy the need of Fe-S biogenesis even during the peak phase of Fe-S protein synthesis. It is worth noting that besides stimulating the cysteine desulfurase activity of NFS2 alike SUFE2 (SufE activity), SUFE3 possesses quinolinate synthase activity which is required for NAD cofactor synthesis (Narayana Murthy et al., 2007). The quinolinate synthase activity of SUFE3 is dependent on the presence of a highly oxygen-sensitive [4Fe-4S] cluster (Cicchillo et al., 2005; Ollagnier-de Choudens et al., 2005; Narayana Murthy et al., 2007). *SUFE3* cannot complement the embryo lethal phenotype of a *sufe1* knock-out mutant, suggesting the SufE activity of SUFE3 is likely dedicated to Fe-S cluster formation in its own quinolinate synthase domain (Narayana Murthy et al., 2007; Balk and Pilon, 2011).

Three mitochondrial proteins are less easily integrated into the general patterns observed here, frataxin (FH), ferredoxin and HSCA2. FH and ferredoxin are thought to have a role in the second step of Fe-S cluster assembly by analogy to human and yeast (Balk and Schaedler, 2014), but their TLs peak at night alongside the TL of several proteins with Fe-S carrier/transfer functions (see Figure 3A, in agreement with Supplementary Figure 1). FH was proposed to deliver Fe to ISU1 by direct interaction with the NFS1/ISD11/ISU1 complex in mitochondria (Balk and Schaedler, 2014). An alternative model has also been discussed in which FH promotes the interaction of the cysteine desulfurase NFS1 with the scaffold ISU proteins to favor the sulfur transfer reaction (Couturier et al., 2013). FH was reported to localize to both mitochondria and plastids (Turowski et al., 2015). HSCA1 can functionally complement the yeast *ssq1* knockout mutant and its ATPase activity is enhanced by HSCB and ISU1 (Xu et al., 2009). The role of HSCA2 in Fe-S protein biogenesis remains to be investigated. Additional and more detailed information will be required to test the possible models arising from this study. In particular also, diel variation in TL was only detected for a subset of proteins in each functional class.

### Extensive Translational Cycling of Fe-S Transfer/Carrier Proteins May Be Determined by Their Specific Roles

Among the 13 Fe-S assembly proteins cycling in translation state, 7 are Fe-S carrier/transfer proteins. ISCA4, NFU4, GRXS15, and ISCA2 localize in mitochondria, whereas NFU1, SUFA, and GRXS14 are localized in plastids. So far, a number of Fe-S transfer/carrier proteins have been identified in *A. thaliana* and they are thought to form a flexible network for Fe-S cluster delivery (Couturier et al., 2013). The function of some Fe-S transfer/carrier proteins may be redundant, because knock-out mutants for most genes encoding Fe-S transfer/carrier proteins are viable (Couturier et al., 2013). For instance, plants deficient in GRXS14 or GRXS16 did not display any growth defect, whereas the growth rate of plants lacking both was reduced (Rey et al., 2017). However, some reports showed that Fe-S carrier/transfer proteins also have specificity for a particular Fe-S target protein or group of targets (Balk and Schaedler, 2014). For instance, NFU2 and HCF101 are involved in the maturation of one or several proteins belonging to PSI and some other stromal proteins (Lezhneva et al., 2004; Stöckel and Oelmüller, 2004; Touraine et al., 2004; Yabe et al., 2004). Thus, we presently cannot decide whether these 7 translationally cycling Fe-S carrier/transfer proteins may preferentially function in the biogenesis of specific Fe-S proteins.

Another feature of Fe-S carrier/transfer proteins is that they act in different manners depending on their redox environment. Mutants for some genes encoding Fe-S carrier/transfer proteins only showed lethal phenotypes specifically under oxygenic conditions. The maturation of two Fe-S proteins in *E. coli*, IspG and IspH, requires a different combination of Fe-S carrier/transfer protein isoforms under different oxygenic conditions. Under aerobic conditions, it uses IscU, IscA and then ErpA for cluster insertion into apo-IspG and apo-IspH, whereas it uses IscU and either ErpA or IscA under anaerobic conditions (Vinella et al., 2009). Another example is cytoplasmic NAR1 in eukaryotes, an Fe-S carrier/transfer protein in CIA pathway. NAR1 forms a complex with CIA1, AE7, and MET18 to transfer Fe-S clusters from the scaffold protein NBP35 to target apoproteins. Interestingly, *nar1* mutants in plants, yeast, and nematodes display phenotypes only under normal oxygen pressure but not under low oxygen pressure (Fujii et al., 2009; Mondy et al., 2014). Therefore, the function of some Fe-S carrier/transfer proteins has a close relationship with the oxygen levels *in* *vivo* and *in* *vitro*, which might explain the existence of extensive cycling of TL of Fe-S transfer/carrier proteins over the diurnal cycle as observed here.

### Open Questions and Prospects

Here we report that the TL of subsets of Fe-S assembly and Fe-S utilizing proteins, as well as of other metalloproteins, vary over a diurnal cycle. It was concluded earlier that Fe-S assembly proteins are not diurnally regulated at the transcript level (Balk and Schaedler, 2014). But there is no investigation so far into whether the rates of Fe-S protein biosynthesis are under circadian or diurnal regulation. Knowledge on the regulation of Fe-S protein biogenesis in plants grown under various regimes of temperature and photoperiod would also help us better understand the mechanisms of *de novo* synthesis or repair of Fe-S clusters (Couturier et al., 2013).

Although much progress was made in elucidating the Fe-S protein biogenesis machinery in plants in the past decades, we are still lacking plant-specific knowledge on the function of a number of predicted Fe-S cluster assembly proteins. Another issue is that the environment-dependent regulation of Fe-S cluster protein biogenesis is largely unknown. Initially, it is important to understand whether and how Fe-S cluster assembly proteins and Fe-S cluster-utilizing proteins are regulated at the transcript, translational and protein levels under various environmental conditions, such as iron and sulfur starvation and other environmental stresses known to enhance internal levels of ROS.

## Conclusion

We assessed the translation state of Fe-S and other metal-dependent proteins genome-wide over a diurnal cycle through the re-analysis of a published microarray dataset. Our analysis suggested that there are notable and complex diel dynamics. Overall, translation state of Fe-S cluster assembly proteins peaked in the middle and at the end of the night (ZT0 and ZT18). Notably, translation state of none of the Fe-S cluster-utilizing proteins peaked during the night, and instead peaked during the day (ZT6 and ZT12). Finally, we observed a trend for peaks in translation state of transcripts encoding proteins requiring different metals at different times of the day. These observations were made only for subsets of proteins of each group. While the biological implications of these observations deserve further study, the diurnal regulation of metalloprotein biogenesis at the level of translational regulation is substantial and should be addressed in a more quantitative and comprehensive study. RNA sequencing-based methods are now available to address this biological question with far higher accuracy (Juntawong et al., 2014).

## Author Contributions

HZ performed all data analysis, wrote first draft of manuscript and edited manuscript. UK conceived study and edited manuscript.

## Conflict of Interest Statement

The authors declare that the research was conducted in the absence of any commercial or financial relationships that could be construed as a potential conflict of interest.
