# Supplementary material for: Differential Diel Translation of Transcripts With Roles in the Transfer and Utilization of Iron-Sulfur Clusters in Arabidopsis
Source: Front Plant Sci. 2018 Nov 13;9:1641. doi: 10.3389/fpls.2018.01641 (PMC6243122; doi:10.3389/fpls.2018.01641)
Supplement: Supplementary file 10 [file Data_Sheet_1.PDF]

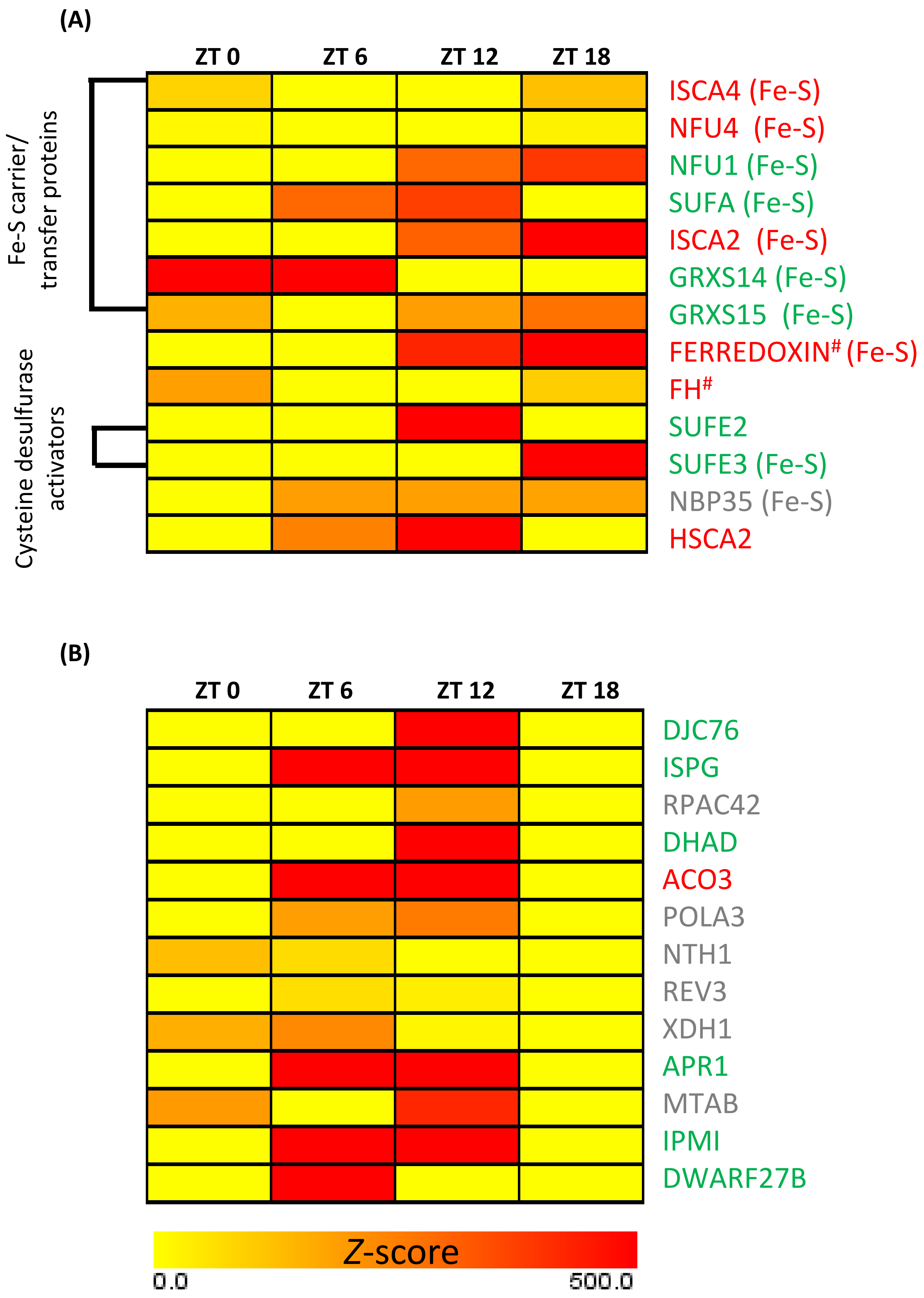

Supplementary Figure 1. Estimated protein biosynthesis rate (PBR) for transcripts encoding Fe-S cluster-related proteins that exhibit significant diurnal variation in translation state. (A) Fe-S cluster assembly, **(B)** Fe-S cluster utilization. PBR data were scaled by the mean center method for each sampling time point and analyzed by software Genesis 1.7.6.
